# Strain-controlled electrophysiological wave propagation alters *in silico* scar-based substrate for ventricular tachycardia

**DOI:** 10.3389/fphys.2024.1330157

**Published:** 2024-04-09

**Authors:** Evianne Willems, Koen L. P. M. Janssens, Lukas R. C. Dekker, Frans N. van de Vosse, Matthijs J. M. Cluitmans, Peter H. M. Bovendeerd

**Affiliations:** ^1^ Department of Biomedical Engineering, Eindhoven University of Technology, Eindhoven, Netherlands; ^2^ Department of Cardiology, Catharina Hospital, Eindhoven, Netherlands; ^3^ Maastricht University Medical Center, Maastricht, Netherlands; ^4^ Philips Research Eindhoven, Eindhoven, Netherlands

**Keywords:** myocardial infarction, functional border zone, longitudinal tissue remodeling, mechanics-driven conduction velocity, long-term risk evolution, phenomenological model, strain amplitudes, virtual-heart simulations

## Abstract

**Introduction:** Assessing a patient’s risk of scar-based ventricular tachycardia (VT) after myocardial infarction is a challenging task. It can take months to years after infarction for VT to occur. Also, if selected for ablation therapy, success rates are low.

**Methods:** Computational ventricular models have been presented previously to support VT risk assessment and to provide ablation guidance. In this study, an extension to such virtual-heart models is proposed to phenomenologically incorporate tissue remodeling driven by mechanical load. Strain amplitudes in the heart muscle are obtained from simulations of mechanics and are used to adjust the electrical conductivity.

**Results:** The mechanics-driven adaptation of electrophysiology resulted in a more heterogeneous distribution of propagation velocities than that of standard models, which adapt electrophysiology in the structural substrate from medical images only. Moreover, conduction slowing was not only present in such a structural substrate, but extended in the adjacent functional border zone with impaired mechanics. This enlarged the volumes with high repolarization time gradients (≥10 ms/mm). However, maximum gradient values were not significantly affected. The enlarged volumes were localized along the structural substrate border, which lengthened the line of conduction block. The prolonged reentry pathways together with conduction slowing in functional regions increased VT cycle time, such that VT was easier to induce, and the number of recommended ablation sites increased from 3 to 5 locations.

**Discussion:** Sensitivity testing showed an accurate model of strain-dependency to be critical for low ranges of conductivity. The model extension with mechanics-driven tissue remodeling is a potential approach to capture the evolution of the functional substrate and may offer insight into the progression of VT risk over time.

## 1 Introduction

Survivors of myocardial infarction have an increased risk of scar-based ventricular tachycardia (VT) (Solomon et al., 2005). VT is an abnormal heart rhythm, where the ventricles beat too fast and lose their pump function. Such arrhythmias can evolve in the months to years after infarction. Clinical markers, such as the left ventricle (LV) ejection fraction, are frequently insufficient to predict a patient’s risk for VT, and the relationship between scar, electrophysiology, and tissue remodeling over time is poorly understood. Consequently, discrimination of post myocardial infarct patients into groups of high and low VT risk can be difficult in the clinic, which results in redundant placement of implantable cardioverter defibrillators. Moreover, for patients selected for invasive treatment, ablation success rates are low.

In literature, a computational heart model is presented to detect patients at risk of VT after myocardial infarction (Arevalo et al., 2016). To assess this risk, personalized simulations of the cardiac electrophysiology are performed, where the infarct area is differentiated from remote tissue in terms of ion channel properties and transverse conduction velocity (CV_T_). In a subsequent study, the model was used as tool to guide invasive treatment by targeting ablation locations (Prakosa et al., 2018). This promising approach to predict VT risk and to provide ablation guidance, is optimized to perform accurate electrophysiological simulations of the heart. However, the evolution of VT risk over time is typically not included in this approach.

It is known that the geometry and material properties of an infarct evolve over time. Machine learning can help to include infarct substrate evolution in computational risk predictions, and it is suggested to repeat model-based risk assessment at follow ups in the clinic to account for remodeling in the diseased area (Shade et al., 2021; Popescu et al., 2022). Still, to our knowledge, there are no genuine longitudinal modeling studies of post-infarct VT performed. Ongoing remodeling can be caused by mechanical load, but in turn also locally alters mechanical load. The effects of mechanics-induced electrophysiological remodeling may play an essential role in explaining the evolution of VT risk over the long timeframe (months to many years) in which post-infarct VTs typically occur. Within this study, we want to explore whether and how changes in mechanical load can alter electrophysiological characteristics and VT risk with it. We focus on mechanical load-induced remodeling of CV_T_, which can be a substrate for VT.

In multiple experiments, a relation between strain and conduction velocity (CV) is shown, from which an overview is given in the review of Jacot et al. (Jacot et al., 2010). Notably, in a series of studies, an increase in CV was observed for higher strain amplitudes in pulsatile stretch experiments with myocyte cultures (Wang et al., 2000; Zhuang et al., 2000; Shyu et al., 2001; Pimentel et al., 2002; Shanker et al., 2005). The CV remained elevated up to hours after the stretching procedure was stopped, which shows the lasting nature of those effects. We assume this permanent effect to be caused by the stain-induced increase in connexin-43, which was observed when strain recured often enough. This is of interest as we noted that connexin expression is linked to slow conduction and VT susceptibility in the healed BZ (Greener et al., 2012). In support of the assumption, a canine study demonstrated that both connexin density and CV in transmural direction increase over the wall depth, with highest values in the last 60% toward the endocardium (Poelzing et al., 2004). This matches with distributions of myofiber-strain, which are highest in endocardial regions as well (Bogaert and Rademakers, 2001; Ashikaga et al., 2007; Ashikaga et al., 2009), further substantiating the idea that repetitive strain, or the lack of it, might affect connexin and CV. The transmural direction in which the CV was measured is perpendicular to the myofiber orientation, and we assume that strain can therefore be linked to CV_T_, which is altered in conventional models as well to reflect the effect of infarction.

**FIGURE 1:**
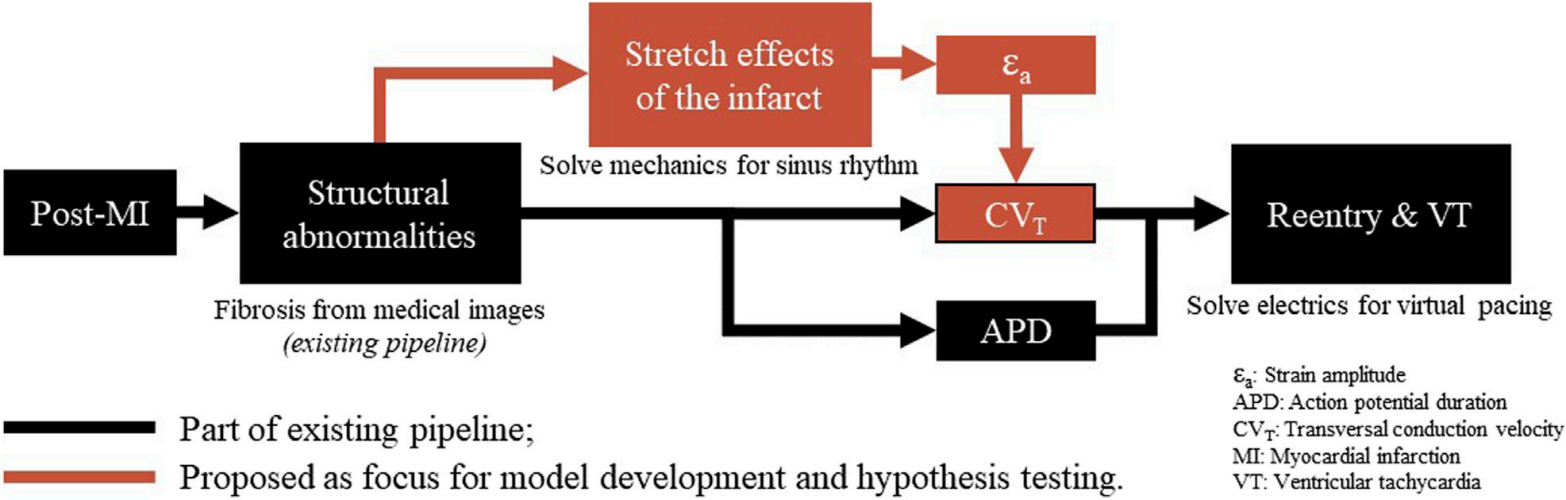
Overview of a virtual-heart model to simulate VT. In the current methods (black), the effect of the structural infarct area is taken into account by altering ion channel properties, which affect the action potential duration, and by reducing CV_T_. In this paper, an extension (red) is proposed to include strain-induced remodeling of CV_T_ as well, which is important for VT risk evolvement over time. Finally, for both models with and without extension, a virtual stress test is performed in an attempt to induce VT.

Our hypothesis is that a link between recurring fiber-strain amplitudes and CV_T_ can be of importance to the development of substrate-based VT, as fiber-strains during the cardiac cycle are affected by stiff fibrosis. We expect that via strain amplitudes, the presence of the infarct affects CV_T_ not only in the infarct core and border zone (BZ), as implemented in conventional heart models, but also in remote tissue parts where mechanics is impaired due to the adjacent structural infarction. Therefore, in this study, we propose a phenomenological model of the relation between fiber-strain amplitude and CV_T_. To test our hypothesis, the phenomenological model proposed is implemented in an existing pipeline (Figure 1). The effects of our hypothesis on electrophysiology simulation outcomes are analyzed in terms of CV_T_ distributions, repolarization time gradients, and virtual stress test results. Finally, the sensitivity of repolarization time gradients to the definition of the strain-CV_T_ relation is explored as well.

## 2 Methods

### 2.1 Model geometry

**FIGURE 2:**
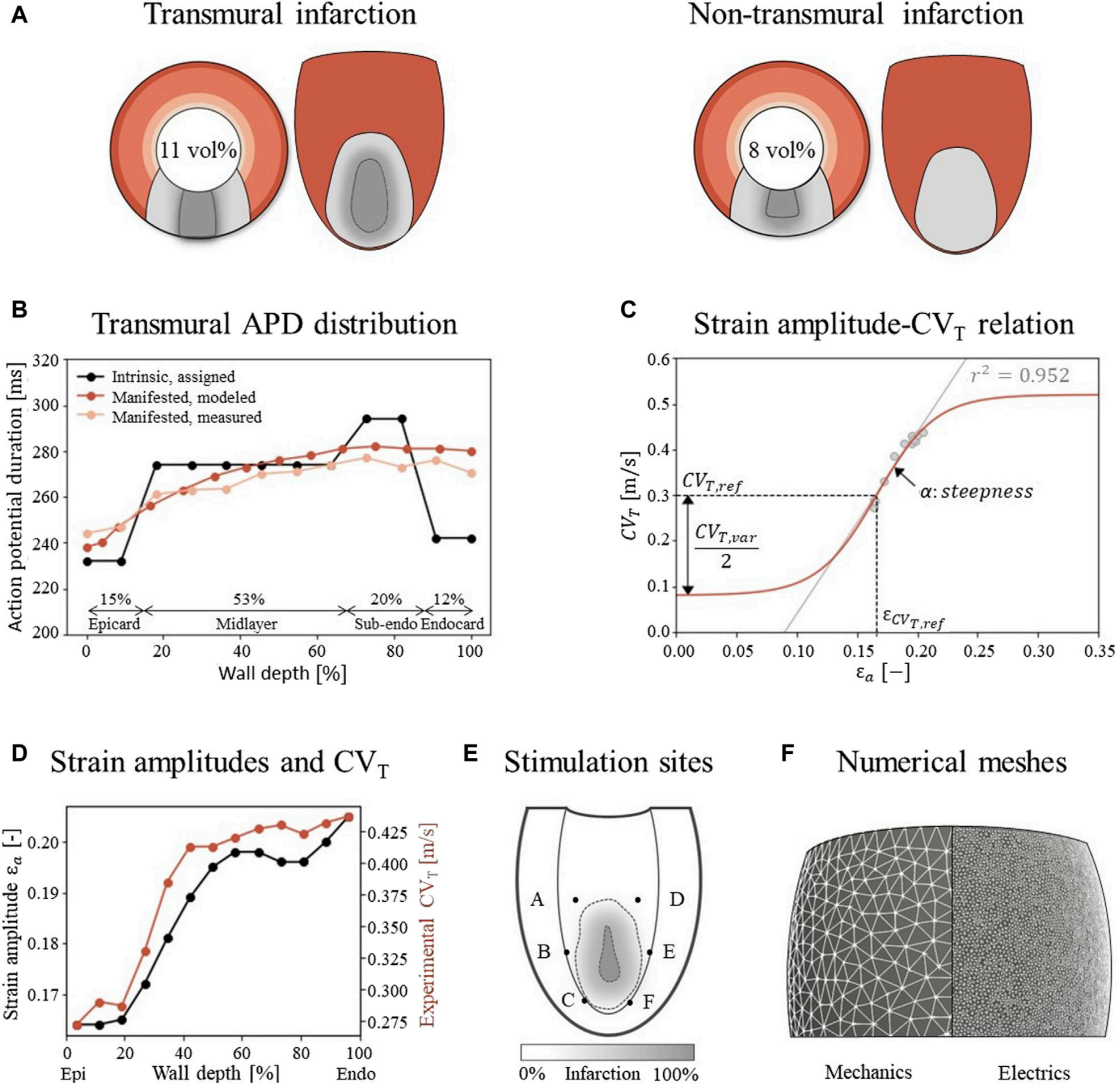
**(A)** Visualization of infarct and BZ in a short axis cross section at ¼ height of the LV, and in a long axis epicardial view. In shades of red, the distribution of different myocardial layers is visualized, as given in subfigure **(B)** In gray, two different combinations of infarct core and structural BZ geometries are schematically depicted, where the latter is a mixture of fibrotic and healthy tissue. The total infarct volume % of the LV is given for both situations. **(B)** For each myocardial layer, a different intrinsic APD is modeled by altering the Gks conductivities to mimic manifested APD measurements as presented by Poelzing et al. (Poelzing and Rosenbaum, 2004). **(C)** Eq. 2.4 (red) describes the phenomenological relation between fiber-strain amplitudes $\varepsilon_{a}$ and CV_T_ when $f_{\mathrm{act}}$ is set to 1. Eq. 2.4 is fitted to a linear regression line through the combined data sets of subfigure D (gray). The meaning of the parameters is visualized in the figure, and corresponding values are given in Table 1. **(D)** Experimental CV_T_ measurements as obtained from Poelzing et al. (red) ((Poelzing et al., 2004) and simulated strain amplitudes $\varepsilon_{a}\left(x\right)$ (black) over LV wall. **(E)** Visualization of six pacing sites at the endocardium. **(F)** Difference in mesh resolution for the mechanical (3 mm) and electrophysiological (500 μm) simulations.

The simulations are performed on a truncated ellipsoidal geometry with a rule-based myofiber field, which represents an idealized LV (Bovendeerd et al., 2009). The wall and cavity volumes are set to 136 and 44 mL. In the ellipsoid, two infarct geometries are considered, where the infarct core extends from 14% to 51% of the ventricle height (Figure 2A). A 100%-transmural core enclosed by a 9 mm thick BZ is implemented to ensure the induction of VTs, as it is known that larger BZs give higher risk for VT. Secondly, an endocardial 67%-transmural core with a 9 mm thick BZ is implemented to investigate whether the extension has the same effect for a non-transmural infarct. The total infarct volumes, including core and BZ, are 15 mL and 11 mL respectively.

### 2.2 Model of cardiac mechanics

The model of Janssens et al. is applied to simulate the mechanical behavior of a chronic infarct ventricle (Bovendeerd et al., 2009; Janssens et al., 2023). The material model and parameter values are copied from this study as well. A brief summary of their methods will be given in this section.

The myocardium is modeled as a non-linear, transversely isotropic, nearly-incompressible material. The Cauchy stress tensor is given by

$$\sigma =f_{pas}\;\sigma_{pas}+f_{act}\;\sigma_{act}{\left(t,l\right)\;\vec{e}}_{f}{\vec{e}}_{f}.$$ (2.1)

This tensor is composed from two parts to account for both passive, $\sigma_{pas}$ , and contractile active, $\sigma_{act}$ , myocardial stresses. $\sigma_{act}$ depends on the time t elapsed since cardiac activation, and on the length l of the contractile element. This active component is only present in parallel with the myofiber orientation ${\vec{e}}_{f}$ . To simulate a chronic infarct, the passive stress $\sigma_{pas}$ is increased by changing the multiplication factor $f_{pas}$ from 1 to 10 in the infarct core to represent stiff fibrosis. Furthermore, the active component is eliminated by setting the multiplication factor $f_{act}$ to 0 to represent the loss of contractile myocytes in this area. To simulate the structural BZ, which is a mixture of infarct and healthy tissue, both $f_{pas}$ and $f_{act}$ values are linearly interpolated from the infarct core to the surrounding healthy tissue, where they are set to 1. The infarct size and geometry differ from those in Janssens et al., and are described in Sec. 2.1.

The ventricle is coupled to a 0D closed-loop lumped parameter model, which represents the circulatory system. Finally, the equation of conservation of momentum is solved:

$$\vec{\nabla }∙\sigma =\vec{0}.$$ (2.2)

Rigid body motion is suppressed by restricting the in-plane solution of basal nodes to its nullspace, which suppresses the average rotation about the long axis as well, and by constraining the base in the out-of-plane direction. The endocardial surface is subject to a uniform pressure. During isovolumetric contraction and relaxation, this pressure is determined such that mechanical equilibrium is obtained at a constant LV volume. The pressure during filling and ejection is computed from the interaction with the circulatory system. The simulated cardiac cycle is initiated by homogeneous activation of the myocardium. Strains are computed by using the LV geometry at t = 0 as reference, and fiber-strain amplitudes are defined as the difference between the local maximum and minimum logarithmic fiber-strain as experienced during the computed cardiac cycle.

### 2.3 Model of cardiac electrophysiology

#### 2.3.1 Reference model

Following the electrophysiology methods of Arevalo et al. (Arevalo et al., 2016), the monodomain equation is used which models cardiac tissue as excitable medium, with diffusion and local excitation of membrane voltage:

$$\beta \left[C_{m}\frac{\partial V_{m}}{\partial t}+I_{ion}\left(V_{m},w,t\right)\right]-\nabla ∙\left(\sigma_{m}\nabla V_{m}\right)=\beta \;I_{ext}\left(t\right)$$ (2.3)

$\sigma_{m}$ is the conductivity tensor describing the effective voltage diffusion through the tissue, $V_{m}$ the membrane voltage which is solved for, β the membrane surface area-to-volume ratio, $C_{m}$ the membrane capacitance, $I_{ion}$ the ionic model which describes the total transmembrane current, w the ionic variables, and $I_{ext}$ is an external stimulus current which can be applied. A zero transmembrane potential flux is imposed on the domain boundary, assuming there is no current flowing into or out of the domain. In this study, β is set to 0.14 μm^-1^, $C_{m}$ to 1 μF/cm^2^, and $I_{ext}\left(t\right)$ to 40 μA/cm^2^ during the delivery of stimuli.

For $I_{ion}$ , the Ten Tusscher ionic model for human ventricular myocytes is used (Ten Tusscher and Panfilov, 2006). The structural BZ is simulated by altering peak conductivities which are kept constant over the whole BZ: for the sodium current (−62%), L-type calcium current (−69%), and potassium currents Gkr (−70%) and Gks (−80%) (Arevalo et al., 2016). This causes an elongation in action potential duration (APD), decreased upstroke velocity and decreased peak amplitude compared to the healthy modeled myocytes. In contrast to the model of Arevalo et al., an intrinsic transmural gradient in APDs is implemented in the remote tissue by altering the peak Gks potassium current in the endocardium (+110%), sub-endocardium (−5%), midlayer (+30%), and epicardium (+145%), yielding intrinsic APDs of 242, 294, 274, and 232 ms respectively when paced with a basic cycle length of 600 ms in single cell simulations. In tissue simulations, this manifests APDs that gradually increase from 238 ms at the epicardium to 282 ms at the endocardium (Figure 2B), which is in agreement with measurements of canine APDs (Poelzing and Rosenbaum, 2004). The gradual increase and maximum difference of 44 ms corresponds with measurements in healthy areas of human hearts (Boukens et al., 2015; Srinivasan et al., 2019). Those manifested (tissue) APDs differ from the intrinsic (isolated cell) APDs due to cell-to-cell communication, also called the electrotonic coupling of cells. The thicknesses of the 4 transmural layers are based upon methods in (Franzone et al., 2006). Those layers and the APD distributions are visualized in Figure 2B.

To simulate electrical pulse propagation, the along-fiber conductivity value for $\sigma_{m}$ is determined to match a CV along the myofibers of 0.6 m/s in remote and BZ tissue. The conductivity is set to 0 in all directions in the infarct core. In the reference model, the methods of Arevalo et al. are followed further by setting CV_T_ (thus in sheet and normal direction) to 0.3 m/s in remote tissue. In the structural BZ, CV_T_ is set to 0.08 m/s and kept constant to represent the effect of a 90% decrease in transverse conductivity (Arevalo et al., 2016). In our extended model, the implementation of CV_T_ deviates from that of Arevalo et al., as explained in the next section.

#### 2.3.2 Extended model

In our new model, we introduce two adjustments to the reference model such that CV_T_ is dependent on both an effect of the structural BZ, and of myofiber strain amplitude. Therefore, CV_T_ is determined by

$${CV}_{T}\left(x\right)={{CV}_{T,ref}\;f}_{act}\left(x\right)\;g\left(\varepsilon_{a}\left(x\right)\right)$$ (2.4)

where ${CV}_{T,ref}$ represents the transverse velocity of 0.3 m/s in the reference model when no effects of scar or strain are taken into account. The first adjustment is that the effect of the structural BZ is not kept constant, but CV_T_ depends on fibrotic density as myofibers can be separated by fibrotic tissue. Through $f_{act}$ as introduced in Sec. 2.2, CV_T_ decreases from its reference value to eventually zero in the infarct core where fibrotic density is highest. The second adjustment is that CV_T_ is affected by a newly proposed phenomenological relation between fiber-strain amplitudes $\varepsilon_{a}$ and CV_T_:

$$g\left({ɛ}_{a}\left(x\right)\right)=1+\frac{{CV}_{T,var}}{2{CV}_{T,ref}}\tanh\left(\alpha \left({ɛ}_{a}\left(x\right)-{ɛ}_{{CV}_{T,ref}}\right)\right)$$ (2.5)

This is a sigmoidal relation (Figure 2C) where the CV_T_ boundaries are set within a physiological range for surviving myocardial tissue. These boundaries are controlled by the allowed variability, ${CV}_{T,var}$ , around the basic velocity ${CV}_{T,ref}$ , which is chosen such that the minimum velocity matches the minimum in the reference model of 0.08 m/s. The function will return 1 if the local strain amplitude equals $\varepsilon_{{CV}_{T,ref}}$ , which will result in a CV_T_ of ${CV}_{T,ref}$ (0.3 m/s) when using Eq. 2.4. The steepness α and shift $\varepsilon_{{CV}_{T,ref}}$ of the relation are chosen to match CV_T_ sensitivity to strain. Their values are determined from combining a canine CV_T_ dataset from literature (Poelzing et al., 2004) with strain amplitudes $\left(\varepsilon_{a}\right)$ obtained from a simulation of healthy LV mechanics (Sec. 2.2). $\varepsilon_{a}$ and CV_T_ are both transmurally sampled in a 2 cm thick slice at the equator of the LV and increase over the wall depth from epi-to endocardium (Figure 2D). This positive correlation (r^2^ = 0.952) is reflected in a positive slope in Eq. (2.5) through α . All parameter values are given in Table 1. For both the reference and extended model CV_T_ is zero in the infarct core, but for the extended model, CV_T_ is expected to be more heterogeneous and to extend beyond the structural BZ into the functional BZ with impaired mechanics, which cannot be detected from contrast enhanced images. To determine CV_T_ values in the electrophysiology model, a weak coupling between the models of mechanics and electrophysiology is established. This is done by obtaining strain amplitudes $\varepsilon_{a}$ and infarct density $f_{act}$ from the model of mechanics, which are then used in Eq. 2.4.

**TABLE 1:** Parameter values in Eq. 2.4 and Eq. 2.5 to describe the relation between fiber-strain amplitude and CV_T_, visualized in Figure 2C.

${CV}_{T,ref}$ [m/s]	0.30	*Basic velocity*
${CV}_{T,var}$ [m/s]	0.44	*CV*_*T*_ *variability*
α [-]	20.6	*Sensitivity constant*
$\varepsilon_{{CV}_{T,ref}}$ [-]	0.1665	*Strain amplitude resulting in* ${CV}_{T}\left(x\right)={CV}_{T,ref}$

#### 2.3.3 Virtual stress test

To evaluate VT risk, a virtual stress test is performed. In such a test, electrical stimuli are applied at six different sites, A through F, around the BZ at the endocardium (Figure 2E) (Kruithof et al., 2021). First, the Ten Tusscher cell model was stimulated 500 times prior to tissue simulations to reach steady-state. The resulting states of gating variables are given as input for the tissue simulations. For these tissue simulations, at each defined site, six stimuli (S1) with a cycle length of 600 ms are applied, followed by a premature stimulus (S2). To explore the vulnerable window in which VTs can be induced, a range in S2 timings with 5 ms increments from earliest electrical capture to 15 ms after the latest induced VT is simulated, resulting in a range from 320 to 370 ms. Each stimulus has a duration of 2 ms, an amplitude of 40 μA/cm^2^ and is applied to a tissue area with a diameter of 3 mm.

### 2.4 Numerical implementation

Unstructured finite-element tetrahedral meshes are generated with an average resolution of 3 mm and 500 μm for the mechanics and electrophysiology model respectively (Figure 2F). Mechanics simulations are performed in FEniCS with timesteps of 2 ms (fenicsproject.org). The displacement field is represented by quadratic Lagrangian basis functions, corresponding to a system of 106,852 degrees of freedom. Electrophysiology simulations are performed in the CARP framework with timesteps of 20 μs (opencarp.org). The membrane voltage potential is approximated by linear Lagrangian basis functions, corresponding to 108,2784 degrees of freedom. To incorporate mechanical results into the electrophysiology simulation, fiber-strain amplitudes are linearly interpolated from the lower resolution mechanics mesh to the higher resolution electrophysiology mesh. From these interpolated strain-amplitudes, CV_T_ values are obtained through Eq. 2.4 and discretized in 9 levels from which the center values are imposed to the extended electrophysiology model using the methods of Costa et al. (Costa et al., 2013).

### 2.5 Experiments performed

The effect of strain-induced CV_T_ remodeling on the electrophysiological simulation outcomes is explored for the two infarct cases as depicted in Figure 2A. For each case, one mechanics cardiac cycle was simulated. Fiber-strain amplitudes from these simulations were used in the extended model to modify the distribution of CV_T_. Virtual stress test simulations from Sec. 2.2.3 are performed for both the reference and extended model to assess the effect of strain-dependent CV_T_.

To investigate the sensitivity of the results to the parameter settings of the novel strain amplitude-CV_T_ relation, the values of α (steepness) and $\varepsilon_{{CV}_{T,ref}}$ (shift) were varied with respect to the baseline values as presented in Table 1 α was set to 50%, 70%, 100%, 150% and 250% compared to the default value. $\varepsilon_{{CV}_{T,ref}}$ was set to 122%, 111%, 100%, 89%, and 78%. This results in even distributions of strain amplitude-CV_T_ curves as shown in the top row of Figure 7, which are applied to the transmural infarct case. For datasets with overall higher measured strains (large $\varepsilon_{{CV}_{T,ref}}$ ), typically a lower sensitivity to variations in strain (small α ) is expected. Therefore, when both parameters are varied, only smaller values for α are considered for larger values in $\varepsilon_{{CV}_{T,ref}}$ .

### 2.6 Evaluation methods

The simulation results are analyzed in terms of the spatial repolarization time gradients (RTG) map. This map is created for the last S1 stimulus in the simulation, where repolarization time is defined as the moment at which the membrane voltage repolarized to −70 mV. From these RTG maps, *RTG*_*vol*_ and *RTG*_*mean*_ metrics are extracted where RTG_vol_ [ml] represents the tissue volume with gradients ≥10 ms/mm, which are critical for conduction block (Restivo et al., 1990). RTG_mean_ [ms/mm] indicates the mean RTG value within this volume with RTGs above 10 ms/mm. These metrics are computed for all performed experiments, such that the effects of mechanical feedback and parameter variability on RTG can be explored. The RTG metrics during baseline rhythm are an indication for VT risk (Cluitmans et al., 2021), and only require S1 stimuli. For the sensitivity test, these metrics are computed for one pacing site only (site A in Figure 2E) to save computational costs.

For the virtual stress test simulations, all six pacing sites are used and S2 stimuli are applied as well. The results are captured in *vulnerable windows*. These windows define for each pacing site and S1-S2 interval whether VT was induced or not. Here, VT is defined as electrical activity sustained for at least 800 ms after S2. In case of occurrence of VT, exit points are defined as the location with earliest second activation time, counted from the start of S2 sustained for at least 800 ms after S2. In case of occurrence of VT, exit points are defined as the location with earliest second activation time, counted from the start of S2.

## 3 Results

### 3.1 Mechanics

#### 3.1.1 Stroke volumes and ejection fractions

**FIGURE 3:**
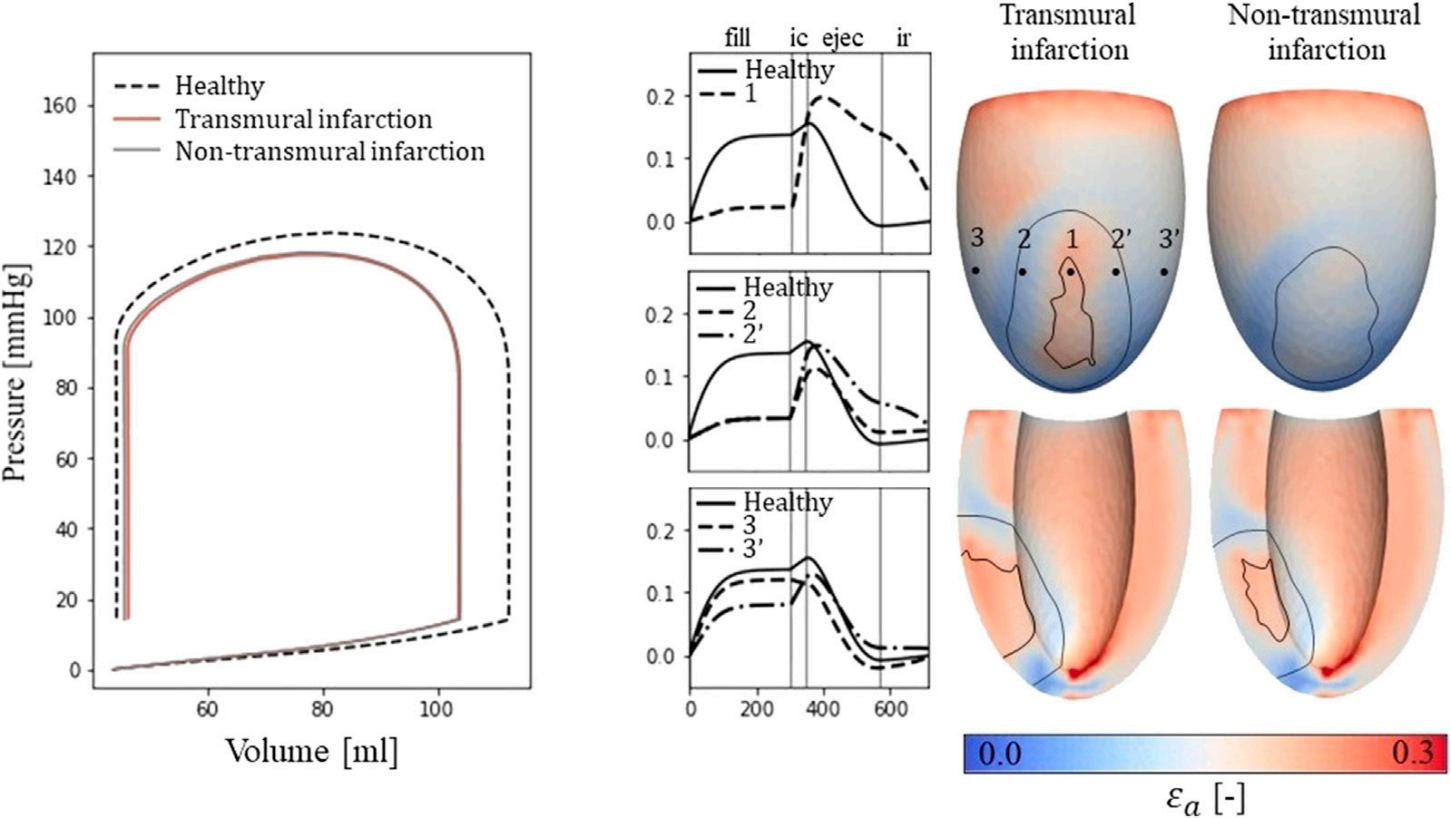
Left: PV-loops as simulated for the healthy LV and the implemented infarcts. Right: myofiber-strain traces during the cardiac cycle where the filling, isovolumetric contraction, ejection, and isovolumetric relaxation phases are indicated with vertical lines. Next to it, spatial distributions of strain amplitudes are given for the infarct cases with an epicardial (top row) and a cross-section view (second row) in which the structural infarct core and border zone are indicated with black lines.

For the simulations of cardiac mechanics, PV-loops are created to capture the global function of the healthy and infarcted ventricles (Figure 3). Stroke volume and ejection fraction for the healthy case are 68 mL and 61% respectively. For the transmural and non-transmural infarcts, this reduced to 57 mL and 55%, and 58 mL and 56% respectively.

#### 3.1.2 Fiber-strain traces and amplitudes

Epicardial fiber-strain traces over time for a healthy case, at the infarct core, the outer BZ, and remote close to the BZ are given in Figure 3 (right-side). For the healthy case, myofiber strain rises during filling and decreases during the ejection phase. At the infarct core (location 1), the fiber-strain shows less increase during filling due to the increased passive stiffness in this area. However, the lack of contractility in this area results in tissue bulging (stretch) during ejection, increasing the fiber-strain during this phase. Therefore, strain amplitudes are still high in the infarct core. For the outer structural BZ (location 2 and 2′), strain during filling is reduced as well as this region is adjacent to the stiff core. Bulging during ejection is less compared to that in the infarct core as active contractile function is mostly retained in the outer BZ, resulting in low amplitudes in and around this region. Finally, in remote tissue close to the BZ (location 3 and 3’), strain traces become more similar to those of the healthy case. Asymmetry in strain results left and right to the infarct is induced by different fiber orientations in those regions relative to the infarct. Overall, strain amplitude is lower at the epicardium compared to the endocardium, in the structural BZ of the infarct, and in parts of remote tissue closely located to the structural BZ.

#### 3.1.3 Left ventricular CV_T_ distributions

**FIGURE 4:**
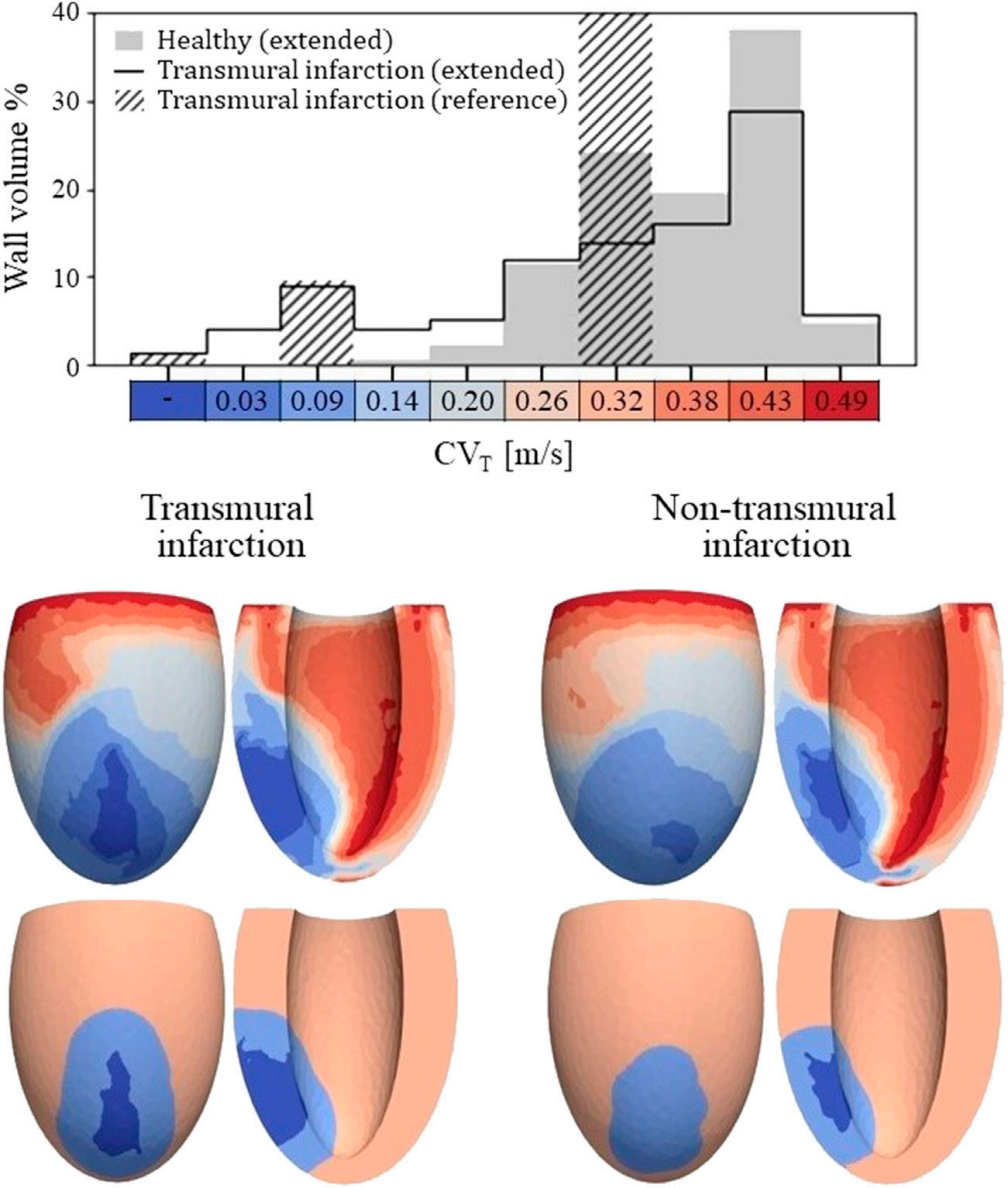
Top: Histogram of CV_T_ values for the healthy and transmural infarct case as obtained with the extended model. The majority of the tissue in the extended models has a CV_T_ in the range of 0.26–0.43 m/s. For the infarct case, velocities below 0.2 m/s are observed, which are not present for the healthy case. Furthermore, CV_T_ for the reference model is given as well, in which CV_T_ is divided over three velocities to represent completely non-viable (first bar), BZ (third bar) and remote tissue (seventh bar). For this reference model, a velocity of 0.3 m/s is assigned to tissue remote from the infarct, which covers 89% of the wall volume (out of frame in the histogram shown). Bottom: Spatial CV_T_ distributions over the LV for the two chronic infarcts, for the extended (top row) and reference model (bottom row). The same discretization and colors are used as for the histogram above.

From the distributions of strain amplitude ( $\left.\varepsilon_{a}\right)$ and scar density ( $\left.f_{act}\right)$ , the LV distributions of CV_T_ are created (Eq. 2.4) for the healthy and two infarct cases. A wider distribution of CV_T_ values is observed for the infarct cases compared to the healthy case (histogram Figure 4), as both $\varepsilon_{a}$ and $f_{act}$ are affected by infarction. For the healthy case, all velocities are within range of 0.20 and 0.49 m/s, where the majority is between 0.26 and 0.43 m/s. For the infarct case, the majority is still within the same range, but conduction slowing induced velocities below 0.20 m/s as well.

The spatial distributions of CV_T_ are shown in the lower half of Figure 4. For both reference (bottom row) and extended (top row) models, conduction slowing is mainly localized at the infarct region. However, for the extended models, this is not limited to the structural BZ but extends to remote parts close to the infarct as well. These regions can be described as functional BZ as they have impaired mechanics due to their connection to the infarct area. Through the model extension, the tissue volume with severe conduction slowing (CV_T_ ≤ 0.1 m/s) increased from 13.0 to 17.5 mL (+35%) for the transmural infarct case. For the non-transmural infarct case, this increased from 10.6 to 17.6 mL (+66%). Furthermore, CV_T_ heterogeneity is present in far remote tissue, where higher values are located towards the endocardium, and rapid CV_T_ changes are present at the apex.

### 3.2 Electrophysiology

#### 3.2.1 Repolarization time gradients

**FIGURE 5:**
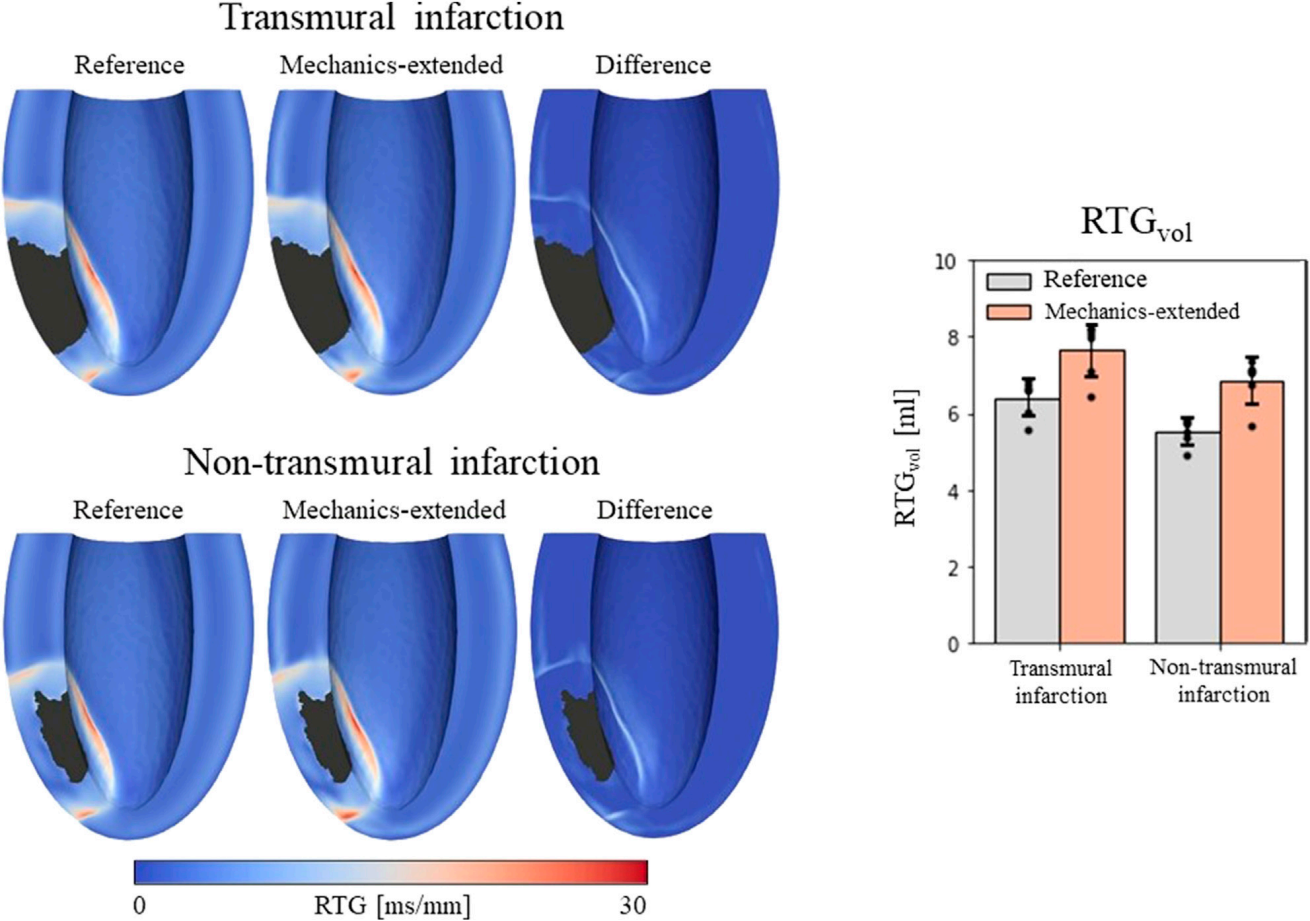
Left: repolarization time gradient maps for the reference model, extended model, and the difference (mechanics-extended minus reference). The extended model further highlights the high gradients on the boundary of the ionic-remodeled border zone. Right: RTG_vol_ and RTG_mean_ per pacing site (dots), with their average values (color bars) and standard deviations (black bars) for both the reference and extended models.

A total of 24 (2*12 settings) RTG maps were created, 2 CV_T_ distributions (reference and extended) for the 2 infarct cases with 6 stimulus sites each. We found that largest RTG values are clustered at the boundary of the structural BZ, in which a prolonged APD was implemented. The mechanics-extension further enhances these high RTG values at the structural BZ boundary (Figure 5, left).

RTG_vol_ and RTG_mean_ values with their average and standard deviations across the 6 stimulation sites are presented in Figure 5 (right-side). The mechanics extension causes RTG_vol_ to increase for all 18 simulation settings. For both infarct cases, this increase in RTG_vol_ is on average 1.3 mL. The mechanics extension has minimal effect on RTG_mean_, which is similar for reference and mechanics-extended simulations, and remains between 12.7 and 14.0 ms/mm for all simulations. RTG_mean_ increased with 0.09 ms/mm for the transmural cases, and with 0.26 ms/mm for the non-tranmsural infarct case. Overall, strain-induced remodeling increased both the volume with severe conduction slowing and RTG the most (dangerous) for the non-transmural infarct case. For a more detailed insight in the effect of the extension on RTG, RTG histograms for the transmural infarct case are given in Supplementary Appendix A.

#### 3.2.2 Virtual stress test

**FIGURE 6:**
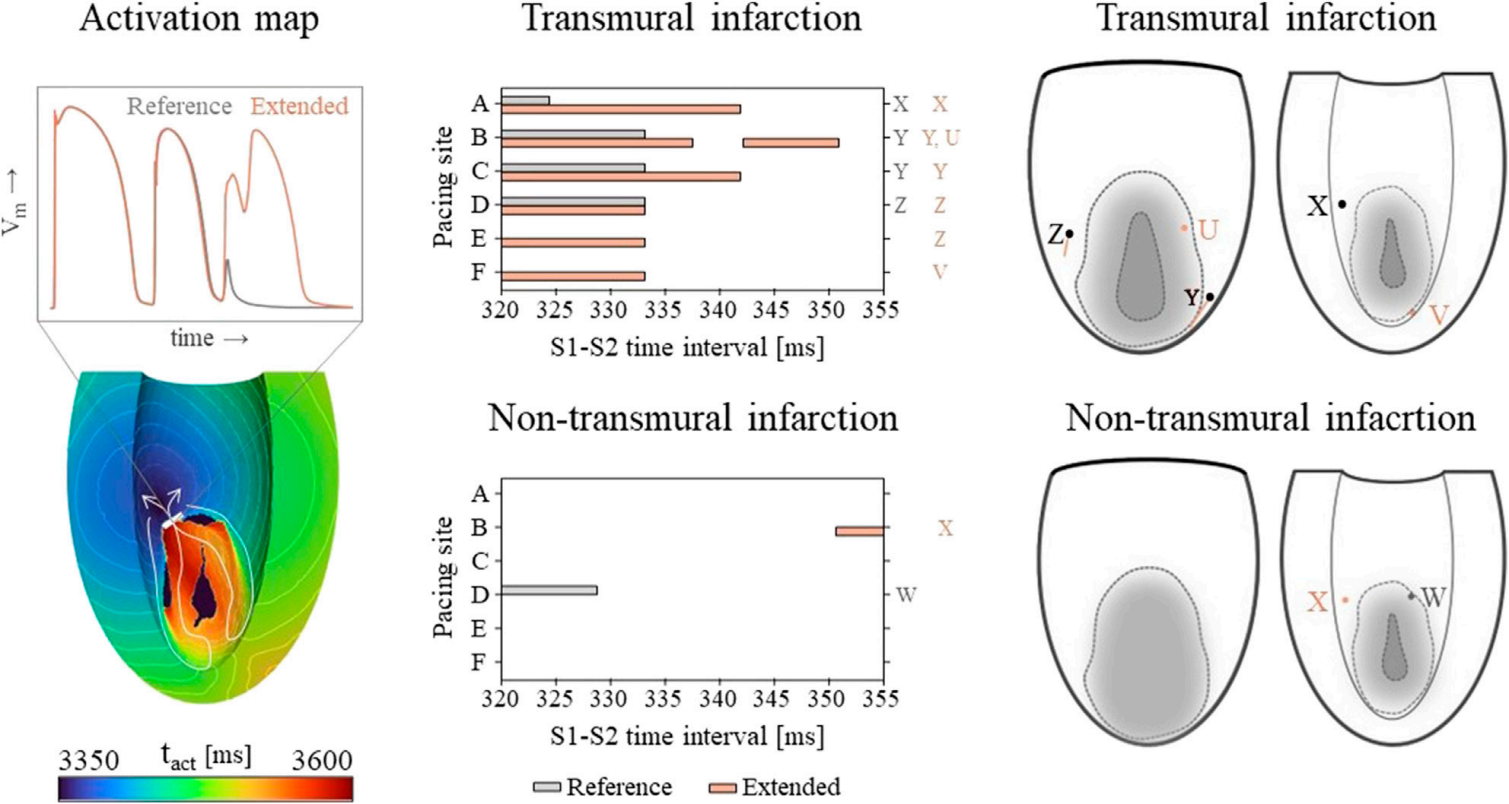
Left: an example of an VT activation times map which shows that VT inducibility can differ for the two models, especially when the effect of the S2 stimulus is on de edge between propagation (white arrow) or conduction block (white block). Middle: vulnerable windows are given with pacing sites A through F on the vertical and S1-S2 time intervals [ms] on the horizontal axis. Color coding indicates whether VT was induced for the reference (gray) or the mechanics-extended model (orange). Characters (U-Z) are used to indicate which windows showed the same VTs. Right: Exit points for each VT pattern are visualized in a schematic representation of the epicardium and endocardium. The assigned characters correspond to those next to the vulnerable windows, and are colored orange if they appeared in the extended model only.

Vulnerable windows for both the reference and extended models are given in Figure 6 (middle). The mechanics-extension has several effects. For the transmural infarct, it more than doubled the vulnerable window width. Importantly, the three VTs as induced in the reference model (labeled X, Y and Z in Figure 6) could be replicated. Their exit sites covered a larger area (orange lines in Figure 6, right-side) compared to the reference results. Additionally, two novel VT patterns (U and V) could be induced. A more detailed analysis showed that the increased inducibility in the extended model was caused by an elongated line of conduction block compared to the reference, due to the enhanced RTGs on the structural BZ boundary. This, together with the additional conduction slowing adjacent to the structural BZ, allowed VT to be more easily sustained. The membrane voltage plot left in Figure 6 demonstrates this finding: the reentrant pathway is not sustained in the reference model, as the tissue was not ready for re-activation when the wavefront arrived too early, in contrast to the extended model due to a slightly longer cycle time.

Despite its high RTG metrics, only small vulnerable windows are present for the non-transmural infarct case (Figure 6, middle). The induced VT patterns differ completely between the reference and extended model. However, their small inducibility windows (5 ms) make them clinically irrelevant.

#### 3.2.3 Sensitivity testing

**FIGURE 7:**
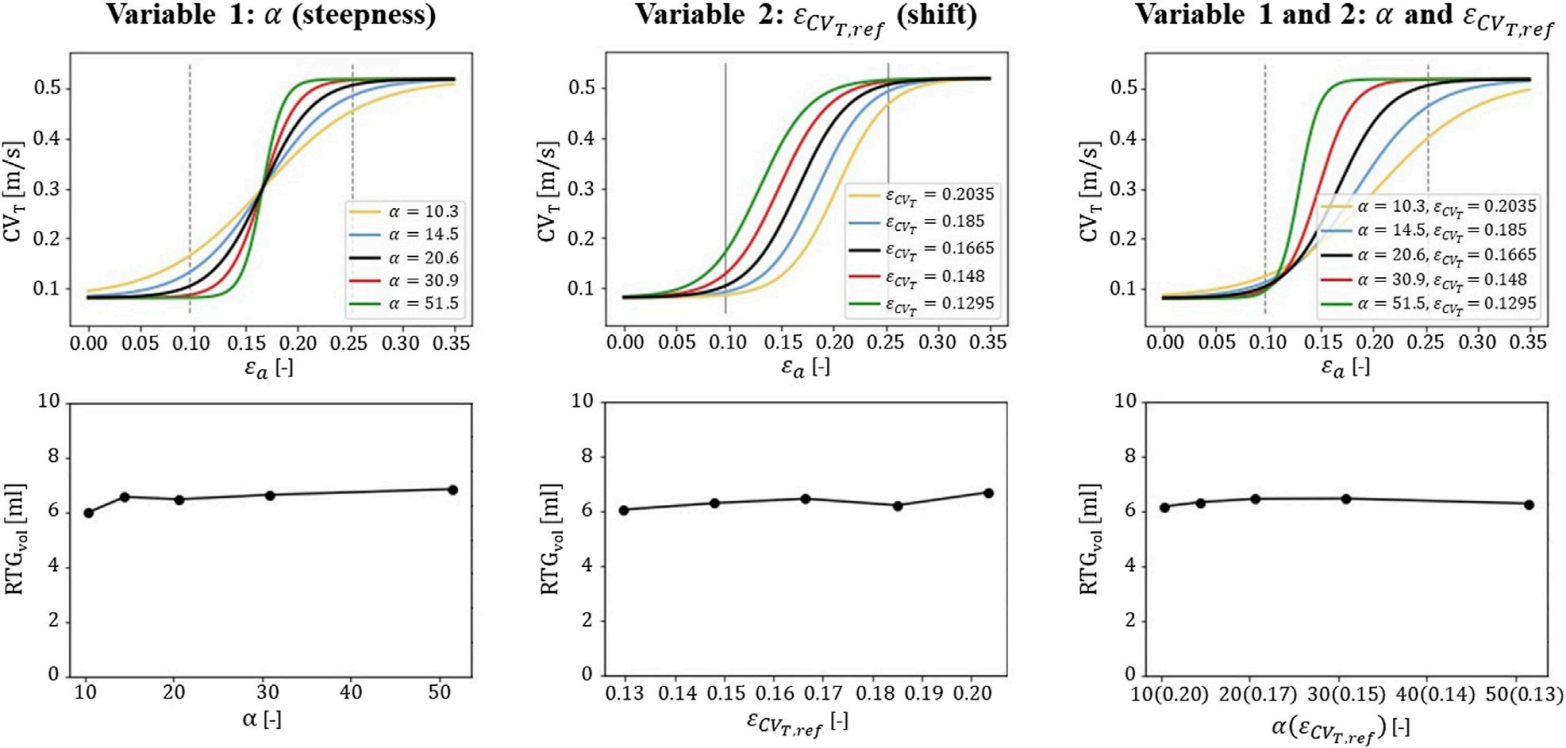
Sensitivity testing of the $g\left(\varepsilon_{a}\left(x\right)\right)$ function. The α and $\varepsilon_{{CV}_{T,ref}}$ parameters are adjusted to obtain homogeneous increments in steepness and shifts of the curve (top row). The corresponding effects on RTG_vol_ is shown for one pacing site (second row). The central values for α and $\varepsilon_{{CV}_{T,ref}}$ (black lines in the top row) are used as default for the stress test simulations (Table 1) for which six pacing sites are used.

The α and $\varepsilon_{{CV}_{T,ref}}$ parameters of the strain amplitude-CV_T_ model (Eq. 2.4; Eq. 2.5) were varied to perform a sensitivity test on the transmural infarct case. This resulted in steepness and horizontal shift variations of the strain amplitude-CV_T_ relational curve as shown in the top row of Figure 7. For each setting, a simulation is performed resulting in a total of 13 RTG maps which are analyzed in terms of RTG_vol_ and RTG_mean_. Within the tested range, the maximal variation in RTG_vol_ (second row of Figure 7) is smaller for altering α (0.9 mL) or $\varepsilon_{{CV}_{T,ref}}$ (0.6 mL) than for altering pacing sites (1.7 mL, right side Figure 5: mechanics-extended, transmural infarction). The variations in RTG_mean_ are minimal. Finally, with the simultaneous increase of α and decrease of $\varepsilon_{{CV}_{T,ref}}$ , variability in RTG_vol_ (0.29 mL) and RTG_mean_ (0.2 ms/mm) is smallest as the curves overlap in the low range of CV_T_, critical for cell-uncoupling and thus RTG. This overlap is also present in histograms of critical RTGs, from which a complete overview is given in Supplementary Appenidx B.

## 4 Discussion

In this study, we explore the hypothesis that chronic changes in mechanical load, expressed in terms of fiber-strain amplitude over the cardiac cycle, affect the transverse electrical propagation velocity and thereby affect VT risk. Therefore, a phenomenological model to describe strain-induced remodeling is implemented as an extension to virtual-heart models from literature.

### 4.1 Methodological modifications

To include the effect of mechanical tissue load, we introduced several modifications to our reference electrophysiology methods. First, in the reference model, CV_T_ is altered in the structural BZ based on measurements in canine infarcts (Yao et al., 2003). Supported by cultured cell experiments, we proposed a phenomenological model in which CV_T_ is altered by recurring strain amplitudes, on top of the effect of structural abnormalities. We found a strong positive correlation (r^2^ = 0.952) between experimental CV_T_ (Poelzing et al., 2004) and simulated myofiber strain amplitude, as they were both lower at the epicardium and higher in the last 60% towards the endocardium. We fitted the model on this correlation, by setting the central CV_T_ to 0.3 m/s as is done conventionally, around which CV_T_ varies due to the effects of strain. This adaptation results in different CV_T_ values for infarct and healthy regions as strain modulates around the stiff infarct.

Second, the effect of fibrotic density on CV_T_ is modelled as a continuous linear relation, instead of the discrete 3-level relation assumed in conventional models. In this way, the highest fibrotic density naturally results in no electrical activity in the non-viable infarct core. Additionally, the spatial CV_T_ distribution is more heterogeneous as it results from the combined effects of fibrosis and fiber-strain amplitudes. These amplitudes are, for example, not constant over the wall depth, and are modulated nearby the stiff infarct. The CV_T_ distribution is therefore discretized in 10 levels instead of the aforementioned 3 (core, structural BZ, and remote) in the reference model.

Thirdly, in order to follow the Poelzing data used in the relationship between experimental CV_T_ and strain, we introduced in our model the transmural APD gradient present in those experimental canine data too. The presence of such transmural APD gradients in human data has been a topic of discussion (Boukens et al., 2015). For completeness, we have performed simulations without the transmural APD gradient too (Supplementary Appendix A). Here we implemented a homogeneous APD (287 ms when paced with 600 ms intervals) over the wall depth. For these simulations, RTGs were lower than for the simulations with transmural APD gradient, and no VTs could be induced. This might be caused by the smaller difference in APD between remote and border zone tissue in this model. Moreover, the occurrence of VT is a binary ‘all-or-nothing’ response, sensitive to various modeling choices. Introducing the effect of strain on CV_T_ might result in VTs in more critical cases. However, the impact of mechanics on electrophysiology substrate is similar under both conditions (with and without transmural APD gradient), enhancing RTGs on the border of the infarct area.

Fourth, we apply a weak coupling between the model of mechanics and electrophysiology. This is motivated from the long time-scale of conduction remodeling observed in cultured cell experiments. This remodeling took a few hours of applied pulsatile stretch, and persisted several hours after the stretch was removed. We assumed that recurring ‘pulsatile’ strains are mimicked during hours of sinus rhythm, which will induce remodeling of CV_T_. These effects would persist long enough to remain in the short timeframe during which stress test pacing is applied. Additionally, the period of pacing is too short to induce further remodeling. It was therefore sufficient to only simulate mechanics during sinus rhythm, from which the results are used to define a static distribution of CV_T_. The numerical uncoupling of mechanics and electrophysiology saves computational costs, and easily allows usage of different simulation environments and mesh compositions for both domains. The downside is that it overlooks instantaneous processes, like the (dynamic) response of strain-sensitive ion channels. However, this was not needed for the focus of our study on tissue remodeling (permanent) over time, in search for underlying mechanisms of VT evolution.

### 4.2 Tissue heterogeneity and VT risk

Our results show a significant effect of mechanical load on the simulation outcome of cardiac electrophysiology. For the healthy LV, transmural variety in fiber strains resulted in heterogeneity in CV_T_ over the ventricular wall depth (Figure 4). The largest strains and, therefore, the highest CV_T_ are found at the endocardium, which is in agreement with experimental results (Poelzing et al., 2004). For the infarcted LV, elevated or reduced fiber-strain amplitudes caused further CV_T_ heterogeneity at the infarct area. It should be noted that this resulted in CV_T_ slowing in remote regions adjacent to fibrotic areas, opposed to conventional models where conduction slowing is limited to structural regions only. Including this functional BZ within regions of impaired mechanics might be a first step in modeling continuous infarct remodeling and expansion.

A second effect of the extension is an increase in tissue volume with severe conduction slowing and in RTG_vol_, which are both substrate metrics. The increase in RTG_vol_ was localized at the structural BZ boundary (Figure 5). We think the lowered CV_T_ adjacent to the structural BZ facilitates cell-to-cell uncoupling on the BZ boundary. This enhances the expression of differences in APD between the structural BZ (longer) and adjacent remote tissue (shorter), increasing RTG on the boundary in between. The elevated RTG caused the lines of conduction block to be longer, resulting in larger reentrant circuits. This, in combination with the lowered CV_T_ in the remote part of reentrant pathways, can prolong reentrant cycle time and more easily result in sustained VT. Those effects might be even more pronounced if cardiomyocyte and APD heterogeneity after infarction is caused by mechanical load as well, as suggested in recent work (Amoni et al., 2023).

LV ejection fraction reduced from 61% in the healthy case to 55% and 56% in the two infarct cases, and did not indicate the difference in VT inducibility. It may show that the LV ejection fraction as current clinical marker is a too global indicator for mechanical function and VT risk (Deng et al., 2016). Our hypothesized strain-induced remodeling might be a way to include local mechanical dysfunction in the assessment of VT risk.

### 4.3 Clinical implications

The hypothesized strain-induced remodeling of CV_T_ might help detect critical regions in the modelled heart. Our extended model could replicate the VT morphologies and their exit sites as identified by the reference model. This is desirable as in previous studies the exit sites as predicted by conventional models and observed retro- and prospectively matched quite well for several swine and human cases (Prakosa et al., 2018). On top of that, novel induced VTs were observed which can lead to identification of additional ablation targets.

The methods presented in this study might be a first step in modeling the effects of pathology induced changes in mechanics on VT risk, which is of interest as post-infarct patients can develop heart failure or dilation. This can alter strain, and a correlation between those diseases, heterogeneous connexin distribution, dispersed impulse conduction, and VT risk is shown (Peters et al., 1997; Kitamura et al., 2002 van Rijen et al., 2004; Boulaksil et al., 2010). However, further experiments should be performed to validate our results and the implemented strain-induced remodeling of CV_T_, as it remains a theoretical hypothesis unproved.

### 4.4 Limitations and recommendations

In this study, the phenomenological model proposed captures the effects of strain on CV_T_, implicitly capturing the role of connexin. Connexin proteins are often assumed to be the link between strain and conductivity, because strain affects connexin expression (Shanker et al., 2005), which in turn affects myocardial conductance (van Rijen et al., 2004; Boulaksil et al., 2010). In future work, explicitly including connexin in a more detailed model might help to obtain additional insight into the process of CV_T_ remodeling over time.

Both our mechanics and electrophysiology models are partly based on animal data. For example, the reference electrophysiology model from literature already included conduction slowing and a prolonged APD in the border zone, which they based on canine data. In our model extension, we had to determine the CV_T_-strain relationship from canine CV_T_ data too. Although we believe our current approach is sufficiently relevant to human conditions to study the potential modulating effects of mechanical strain on conduction velocity, this species mismatch should preferably be addressed in future studies.

Strain data from endo-to epicardium in literature is divers (Ashikaga et al., 2007; Bogaert and Rademakers, 2001; Costa et al., 2013; Takayama et al., 2002), which results in uncertainty in the accuracy of the relationship between strain and CV_T_ proposed. Therefore, we investigated the effect of parameter variation in the extended model. In particular, we focused on parameters that alter the steepness and shift of the relation proposed (Figure 7). We found the definition of the relation for low strain amplitudes (˂0.12) to be critical. Relational changes for this range affected RTG results the most, because those low amplitudes are found in and around the BZ, where they determine the amount of conduction slowing. This finding stresses that further research is needed to investigate whether the proposed relation correctly describes strain-induced remodeling of CV_T_, especially for lower strain amplitudes. Further improvements may be obtained with personalized models, which allows for validation by linking patient-specific strain-imaging data.

In the model of mechanics, chronic infarct areas were implemented by increasing the stiffness as a result of fibrotic tissue, and by reducing contractility due to a loss of myocytes. However, contraction was initiated simultaneously throughout the LV, neglecting asynchronous electrical activation. As a next step, this can be included which would allow simulation of a feed forward cycle to describe post-infarct tissue evolution. Altered strains around the infarct may affect the electrical conductivity, which will affect mechanical activation in turn and therefore further modulate the strain. This cycle can then be repeated. Such a feed forward cycle is not yet included.

Finally, in this work, only one mechanics cardiac cycle was simulated which is not enough to reach hemodynamic steady state. To investigate the consequences, we performed a convergence study in which we ran the model for transmural infarct for 5 subsequent cycles. On top of that, both the mechanics and electrophysiology mesh was refined, and the number of CV_T_ discretization levels was doubled. Strain amplitudes remained similar in remote areas while low strain amplitudes in the BZ region (±0.15) further reduced by up to 0.02. The spatial CV_T_ distribution remained similar and CV_T_ in the BZ reduced maximally with 0.06 m/s. The lowered amplitudes and CV_T_ further enhance the effect of the mechanics extension, and the effect on the critical RTG substrate remained the same (Supplementary Appendix A).

## 5 Conclusion

We hypothesized that transverse myocardial conduction velocity alters with recurring myofiber strain amplitude. In a model of cardiac electromechanics, this hypothesis led to transverse conduction slowing in the structural BZ, successfully replicating its abnormal electrophysiological behavior. On top of that, conduction slowing was heterogeneous and extended into tissue regions adjacent to the infarct area, with impaired mechanical function. This ‘functional’ BZ fostered accumulation of high repolarization time gradients along the structural substrate boundary. In a stress test, it led to an increase of the vulnerable window of occurring VTs, and additional VT patterns. Our hypothesis might be a first step to consider the effects of pathology induced changes in mechanical diseases in silico VT-risk estimations. However, further experiments should be performed to investigate the accuracy of the relationship between strain and conductivity proposed.

## Data Availability

The original contributions presented in the study are included in the article/Supplementary Material, further inquiries can be directed to the corresponding author.
