# Supplementary material for: Strain-controlled electrophysiological wave propagation alters in silico scar-based substrate for ventricular tachycardia
Source: Front Physiol. 2024 Apr 9;15:1330157. doi: 10.3389/fphys.2024.1330157 (PMC11036413; doi:10.3389/fphys.2024.1330157)
Supplement: Supplementary file 1 [file DataSheet1.docx]

**Appendix A:** RTG analysis of the transmural APD gradient and convergence test simulations


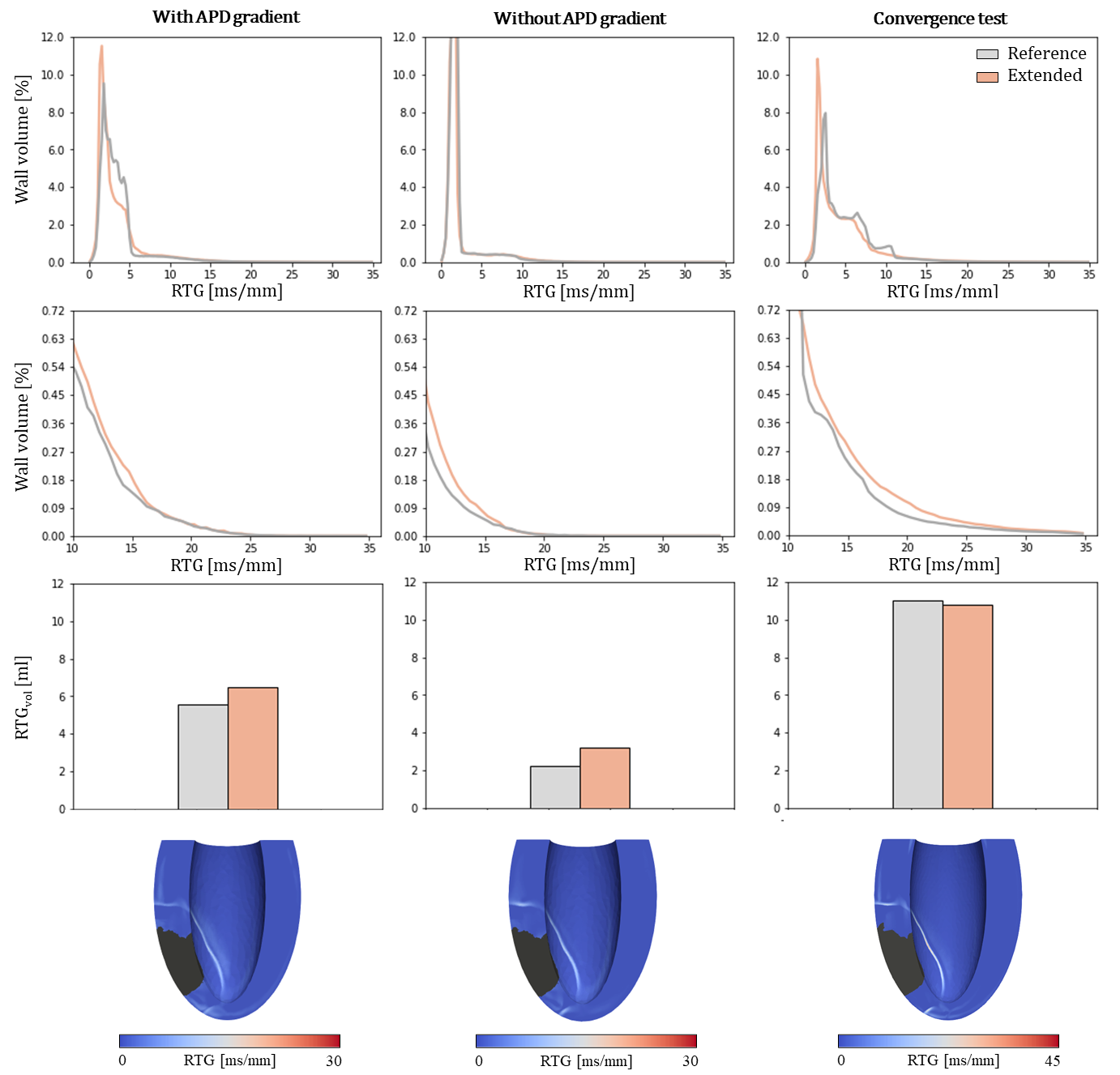


*Figure S1. RTG analysis corresponding to simulations with transmural APD gradient (left), without transmural APD gradient (middle), and to convergence test simulations with transmural APD gradient using refined mesh resolutions, hemodynamic steady state, and refined CV_T_ discretization (right). For each case, results are given in RTG histograms, a zoom-in of those histograms to RTGs relevant for VT risk (>10 ms/mm), the RTG_vol_ metric, and a spatial map of the difference in RTG values between a simulation with and without the effects of mechanics. The latter is calculated as RTGs for the mechanics-extension simulations minus those for reference simulations. The histogram x-axis gives RTG values, and the y-axis the LV wall volume percentage in which these RTG values are found. The peak with low RTG values in the top row is found in remote tissue. Those RTGs are (left) slightly reduced by the mechanics-extension as it reduces differences in repolarization times due to an enhanced conductivity and thus electrotonic coupling in remote areas. This effect is not found for simulations without transmural APD gradient (middle): the changed conductivity and thus electrotonic coupling does not affect repolarization differences in the remote area, as the differences are already minimal due to the homogenous APDs. In the close-up histograms can be seen that the effect of the mechanics extension on critical RTGs is the same for all tests performed. This is confirmed in the spatial difference maps, where the bright color shows that the mechanics-extension enhances RTG values on the border of the structural infarct area. However, absolute RTG values increased with increasing mesh resolution (right, note the different legend scale for the spatial map). Therefore, the effect of mechanics could not be captured with the RTG_vol_ metric as the threshold of 10 ms/mm used is too low for this case, capturing remote tissue areas as well with values between 10 and 11 ms/mm due to the shift in RTGs, and does not represent the effect of mechanics observed in the spatial map.*

**Appendix B:** RTG analysis for the sensitivity tests


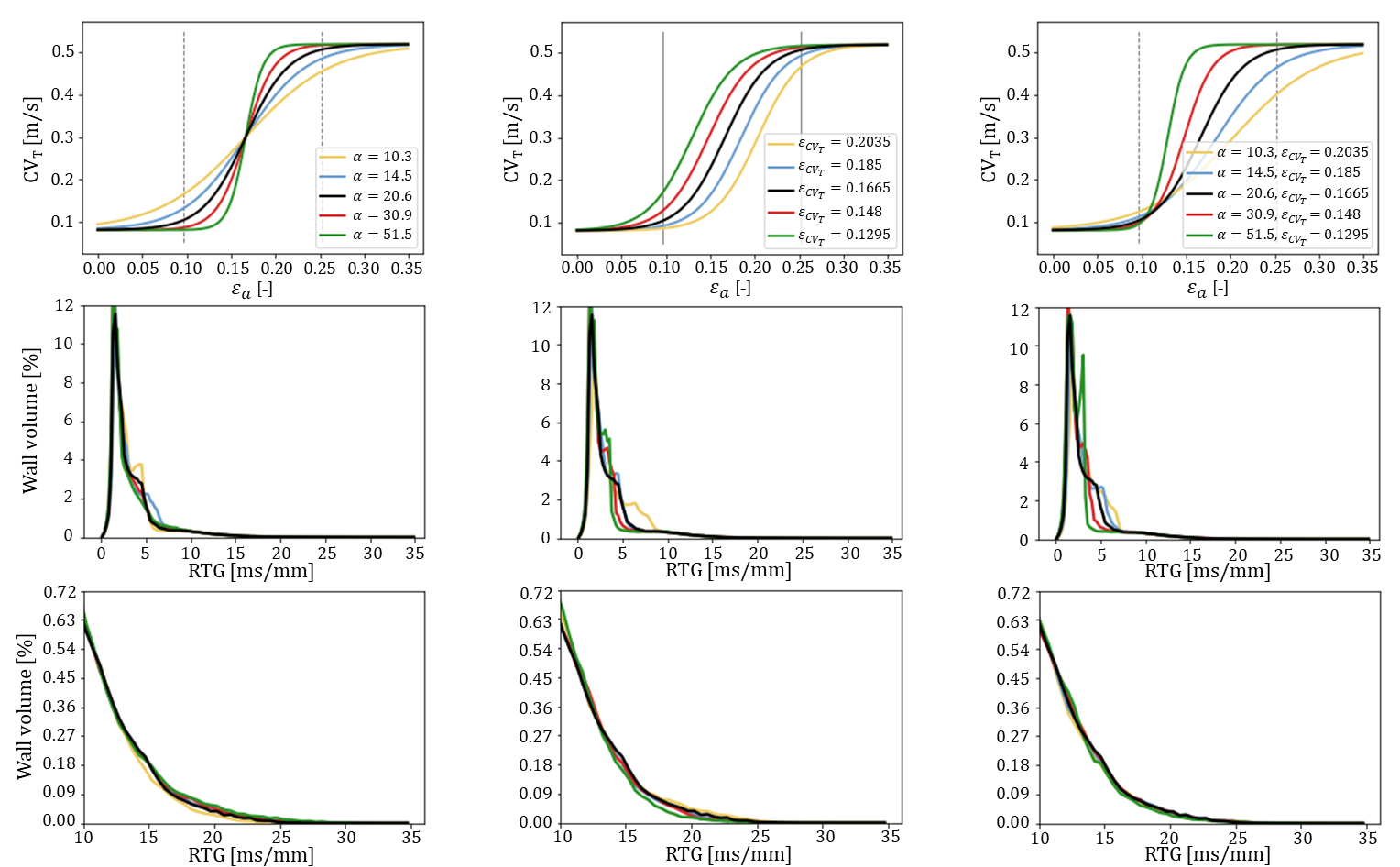


*Figure S2. RTG analysis for the sensitivity tests (Sec. 3.2.3). The top row shows the parameter variations used for the strain-CV_T_ relation. The second row shows RTG histograms, and the last row a close-up of the critical range in RTG values (> 10 ms/mm) of these histograms. This close-up confirms that the critical RTG regions are not sensitive to the parameter variations applied.*
